# circITGA7 Acts as a miR-370-3p Sponge to Suppress the Proliferation of Prostate Cancer

**DOI:** 10.1155/2021/8060389

**Published:** 2021-12-31

**Authors:** Gang Luo, Guohao Li, Zhihua Wan, Yuanjie Zhang, Dong Liu, Yonglian Guo

**Affiliations:** ^1^Department of Urology, The Central Hospital of Wuhan, Tongji Medical College, Huazhong University of Science and Technology, Wuhan, Hubei 430014, China; ^2^Department of Urology, Shanghai Pudong Hospital, Fudan University Pudong Medical Center, Shanghai 201399, China

## Abstract

Prostate cancer (PCa) refers to one of the most common tumors in male's genitourinary system. Emerging research has confirmed that circRNAs play an important role in the occurrence and development of tumors. However, the correlation between circular RNA circITGA7 and PCa still remains unclear. Here, the role of circITGA7 in PCa was explored and the underlying mechanism was investigated as well. The circRNA expression profiles in PCa and the paracancerous tissues were established by high-throughput sequencing. The expression levels of circITGA7 in PCa tissues and cells were detected by qRT-PCR. Cell Counting Kit-8, colony formation, EdU, and flow cytometry assays were used to detect the effects of circITGA7 on PCa cell proliferation. To further explore the underlying mechanisms, bioinformatics analysis on downstream target genes was carried out. RNA immunoprecipitation and dual-luciferase reporter assays were used to verify the direct relationship between miR-370-3p and circITGA7 or P21^CIP1^. The present results demonstrated that circITGA7 was downregulated in PCa tissues and cells. Gain- or loss-of-function assays showed that circITGA7 inhibited the proliferation of PCa cells *in vivo* and *in vitro*. Mechanically, circITGA7 served as a sponge for miR-370-3p, and miR-370-3p could target P21^CIP1^ in PCa cells. The inhibition of cell proliferation induced by circITGA7 could be reversed by transfecting miR-370-3p mimic. Collectively, our data indicated that circITGA7 played an important role in inhibiting tumor proliferation in PCa and might be a potential therapeutic target.

## 1. Introduction

Prostate cancer (PCa) is the second most common cancer in men worldwide, with high morbidity and mortality [1]. Although localized PCa can be cured by surgical resection, many patients were diagnosed with distant metastases and missed the opportunity for surgery. Androgen deprivation therapy (ADT) is the first choice for the treatment of metastatic PCa and can rapidly inhibit tumor progression in the short term. However, almost all PCa patients treated with ADT will develop into hormone resistance prostate cancer (CRPC) and die within 2–4 years [2]. Therefore, it is urgent to better understand the pathogenesis of PCa and find new therapeutic targets.

Circular RNA is a new type of noncoding RNA without 5′ cap and 3′ tail. It forms a closed circular structure by back-splicing mechanism and is more stable than linear noncoding RNA [3, 4]. First identified under the electron microscope in 1979, circRNAs were initially thought as the products of precursor mRNA splicing errors [5, 6]. With the advent of high-throughput sequencing, it has been found that circRNAs are widely expressed in eukaryotes [7]. Furthermore, increasing evidence revealed that circRNAs played an important role in the development of malignant tumors, including PCa [8–10].

circITGA7, generated from the exon 4 of integrin subunit alpha 7 (ITGA7) gene by back-splicing, was first reported to inhibit colorectal cancer growth and metastasis by Li et al. in 2018 [11]. Subsequently, its regulatory role in thyroid cancer, osteosarcoma, glioma, and gastric cancer was reported successively [12–15]. However, the expression and biological role of circITGA7 in PCa are still unclear. In this study, we identified the circRNAs differentially expressed in PCa based on high-throughput sequencing and found that the expression of circITGA7 in PCa tissues was significantly lower than that in paracancerous tissues. We further examined the effects of circITGA7 on the proliferation of prostate cancer cells by CCK-8, EdU, colony formation, and flow cytometry and explored the underlying mechanisms. Our study may provide a potential new target for the diagnosis and treatment of PCa. A preprint of our work has previously been published in Research Square (DOI: 10.21203/rs.3.rs-726944/v1) [16].

## 2. Materials and Methods

### 2.1. Tissue Samples and Cell Lines

A total of 28 pairs of PCa samples and corresponding adjacent normal prostate tissues were obtained from patients who underwent radical resection of prostate cancer at the Central Hospital of Wuhan between 2016 and 2019. A summary of clinical data is listed in Supplementary Table 1. Patients who had received chemotherapy, radiation therapy, endocrine therapy, or other anticancer treatments prior to surgery were excluded from our study. The research protocol was approved by the Ethics Committee of the Central Hospital of Wuhan, and informed consent was obtained from each patient before enrollment.

Human prostate cancer cell lines (DU145, LNCaP, PC3) and normal human prostate epithelial cells (RWPE-1) were acquired from the Institute of Cell Research, Chinese Academy of Sciences (Shanghai, China). Cells were incubated at 37°C and 5% CO_2_ in DMEM medium (Gibco, USA) containing 10% FBS (HyClone, USA) and 1% penicillin/streptomycin (Gibco, USA).

### 2.2. RNA-Seq Analysis

High-throughput sequencing was performed in three pairs of prostate cancer and paracancerous normal tissues, and the samples were processed and sequenced by Kangce Technology Co., Ltd (Wuhan, China). The results were processed with the edgeR software package (version 3.12.1). *P* value cutoff of 0.05 and fold-change cutoff of 2 were used to judge the statistical significance of gene expression differences. The RNA-seq data were deposited at the NCBI Gene Expression Omnibus database (https://www.ncbi.nlm.nih.gov/geo/query/acc.cgi?acc=GSE179321) with accession number GSE179321.

### 2.3. RNA Purification and qRT-PCR

Total RNAs from cells and tissues were extracted using TRIzol Reagent (Invitrogen, USA) according to the manufacturer's instructions. RNase R treatment was processed at 37°C with 3 U/mg of RNase R (Epicenter, USA) for 15 min. Complementary DNA was synthesized using the PrimeScript RT Master Mix (Takara, China) or the miScript Reverse Transcription Kit (Qiagen, Germany) from 500 ng of RNA with random or oligo (dT) primer. The quantitative real-time PCR analyze (qRT-PCR) was conducted using SYBR Premix Ex Taq™ kit (Takara, China) or a miScript SYBR Green PCR Kit (Qiagen, Germany). The values of 2^−ΔΔCT^ relative to one of the samples were calculated to analyze relative expression levels of the RNAs. GAPDH or U6 was used as the endogenous control for mRNA and circRNA or miRNA, respectively. All the primers are listed in Supplementary Table 2.

### 2.4. Plasmids Construction and Transfection

To artificially upregulate or downregulate the expression of circITGA7, the pcD-ciR overexpression vector (circITGA7), empty vector (vector), small interfering RNA targeting circITGA7 (si-circITGA7), and negative control (si-NC) were purchased from GenePharma (Shanghai, China). miR-370-3p mimic and its corresponding negative control (mimic-NC) were purchased from RiboBio (Guangzhou, China). All the vectors were transfected into cells with Lipofectamine 3000 (Thermo Fisher Scientific, USA) following the manufacturer's instructions.

### 2.5. Cell Counting Kit-8 Assay

At 48 h posttransfection, 1 × 10^4^ cells were plated into 96-well plates and then incubated with 5% CO_2_ at 37°C for 24, 48, 72, or 96 h. Then, 10 *μ*L CCK-8 solution (Sigma-Aldrich, USA) was added in each well, and the cells continued to be cultured for an additional 2 h. The absorbance was measured at 450 nm using a microplate reader.

### 2.6. Colony Formation Assay

For colony formation assay, stably transfected cells were seeded into six-well plates with 1000 cells per well and cultured for 2 weeks at 37°C with 5% CO_2_. The colonies were fixed in methanol and stained with 0.1% crystal violet. Cell colonies with more than 50 cells were counted.

### 2.7. EdU Assay

The EdU assay was carried out using Cell-Light EdU DNA Cell Proliferation Kit (RiboBio, China) according to the manufacturer's protocol. At 48 h posttransfection, cells (1 × 10^5^ cells/well) were seeded in 96-well plates, and EdU (100 mmol/l; RiboBio) was added to the medium and incubated for 2 h at room temperature in the dark. Then, 100 *μ*l Hoech st 33342 (2 *μ*g/ml; RiboBio) were used to stain the DNA contents of the cells. Images were obtained with an Olympus FSX100 microscope (Olympus, Japan). The ratio of EdU-stained cells to Hoechst-stained cells was calculated to evaluate the cell proliferation.

### 2.8. Fluorescence In Situ Hybridization

Cy3-labeled circITGA7 were designed and synthesized by RiboBio (RiboBio, China). These probes were mixtures, and their sequences were not publicly available. Cells were firstly fixed in 4% paraformaldehyde and incubated with 0.1% Triton X-100 on ice for 10 min. 2.5 *μ*l circITGA7 probes (20 *μ*M) were hybridized with the cells for 5 h in the dark at 37°C. DAPI was used to stain the nucleus. The images were acquired on Nikon A1Si Laser Scanning Confocal Microscope (Nikon Instruments Inc, Japan).

### 2.9. Flow Cytometry Analysis of Cell Cycle

Cells transiently transfected with circITGA7 or si-circITGA7 were harvested and stained with propidium iodide buffer (Thermo Fisher Scientific, USA) for 15 min. Cell cycle analysis was performed by FACScalibur flow cytometer (BD Pharmingen, USA). The results were analyzed by the ModFit LT software.

### 2.10. Dual-Luciferase Reporter Assays

Wild-type and mutant sequences of circITGA7 and P21^CIP1^ 3′-UTR were synthesized by Tsingke Biological Technology Co., Ltd., and cloned into pGL3 promoter vector by RiboBio (RiboBio, China). Cells were seeded at a density of 1 × 10^4^ into 96-well plates. Then, the luciferase vectors were transfected into cells together with miR‐370-3p mimic or mimic-NC, respectively. Luciferase activities were determined by a dual‐luciferase reporter assay kit (Promega, USA).

### 2.11. RNA Immunoprecipitation

The EZMagna RIP kit (Millipore, USA) was used to evaluate the target relationship between miR-370-3p and circITGA7. Cells were harvested and lysed in RNA lysis buffer containing RNase and protease inhibitors on ice for 5 min. Then, cell lysates (100 *μ*l) were incubated with 40 *μ*l protein A/G beads and 5 *μ*g human anti-Ago2 antibody (Millipore, USA) or 5 *μ*g IgG (Millipore, USA) overnight at 4°C. Next, the RNA/bead complexes were washed with RIP wash buffer and resuspended in Proteinase K buffer on a shaker at 58°C for 30 min to separate proteins. The immunoprecipitated RNAs were used for identification by qRT-PCR assay.

### 2.12. Western Blot Assay

Cells were collected at 48 h posttransfection and resuspended in RIPA lysis buffer (Beyotime, China). In total, 50 *μ*g of each protein sample was separated by SDS-PAGE and then transferred onto polyvinylidene difluoride membranes (Millipore, USA). After being blocked with 5% blocking buffer, the membranes were incubated with P21^CIP1^ antibodies at 4°C overnight. ECL chromogenic substrate (Beyotime, China) was used to visualize the bands. The antibodies against P21^CIP1^ and GAPDH were purchased from Abcam (Abcam, USA).

### 2.13. Tumor Xenografts Experiments

PC3 cells (3 × 10^6^, 200 *μ*l) stably transfected with circITGA7 or control vector were subcutaneously injected into male BALB/c nude mice (4 weeks old). Then, the mice were maintained for three weeks before being sacrificed by cervical dislocation. The tumor volume was recorded every 3 days. The tumor volume was calculated according to the following formula: volume = length × width^2^/2.

### 2.14. Statistical Analysis

Data were presented as mean ± standard deviation. Statistical analysis was performed using GraphPad Prism 7.0 (La Jolla, USA). All experiments were independently repeated in triplicate. The *χ*2 test was applied to determine the associations between circITGA7 expression and the clinical parameters. Pearson correlation analysis was used to analyze correlation between the expression of circITGA7 and miR-370-3p. Comparison between tumor and adjacent noncancerous tissues was analyzed using paired Student's *t*-test. Comparison between two independent groups was analyzed via unpaired Student's *t*-test, while comparisons among multiple groups were calculated by one-way ANOVA followed by Tukey's test. *P* < 0.05 was considered to indicate a statistically significant difference.

## 3. Results

### 3.1. circITGA7 Is Downregulated in PCa Tissues and Cell Lines

**Figure 1:**
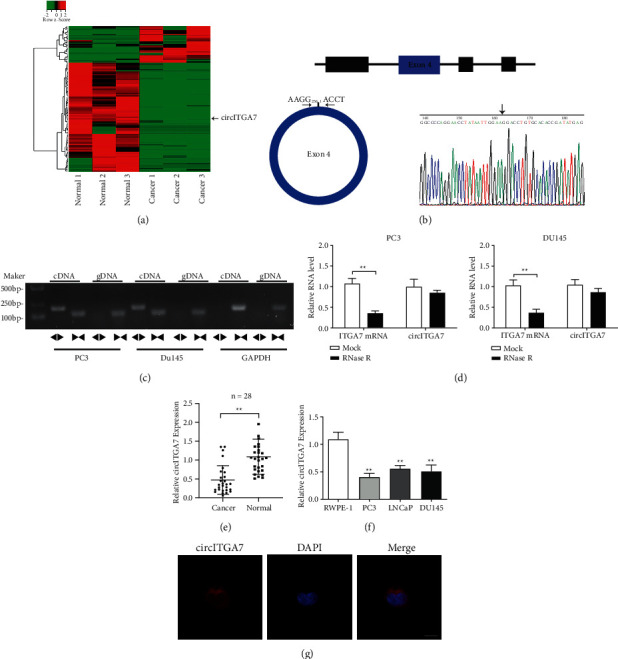
Relative circITGA7 expression levels in PCa. (a) Clustered heat map of the differentially expressed circRNAs in three pairs of human PCa tissues and adjacent normal tissues (GSE179321). (b) Schematic illustration shows the circularization of ITGA7 exon 4 formed circITGA7. The back-splice junction of circITGA7 was identified by Sanger sequencing. (c) PCR assay with divergent or convergent primers indicated the existence of circITGA7 in PCa cells. Divergent primers ◀▶; convergent primers ▶◀. (d) The expression of circITGA7 and ITGA7 mRNA in PCa cells treated with RNase R was detected by qRT-PCR. (e) The expression of circITGA7 in 28 pairs of PCa tissues and paracancerous tissues was detected. (f) The relative expression of circITGA7 was detected using qRT-PCR in RWPE-1, PC3, LNCaP, and DU145 cells. (g) RNA-FISH indicated the location of circITGA7 in PC3 cells. Scale bar, 10 *μ*m. Data were presented as mean ± SD of three independent experiments. Statistically significant differences were indicated:  ^*∗*^ ^*∗*^*P* < 0.01.

To identify the circRNAs differentially expressed in PCa, we performed RNA sequencing in three pairs of prostate cancer and paracancerous tissues, and the results illustrated that 29 circRNAs were significantly upregulated and 87 circRNAs were obviously downregulated in PCa tissues compared with paracancerous tissues (Figure 1(a)). The differential expression of circITGA7 in tumors and its ability to participate in tumor development have been reported but its role in PCa remains unclear, so we chose to conduct further studies on circITGA7. The genomic structure indicated that circITGA7 was generated from the exon 4 of the ITGA7 gene and the head-to-tail splicing of circITGA7 was further confirmed by Sanger sequencing (Figure 1(b)). Afterwards, we designed convergent primers and divergent primers to amplify linear and circular RNA on the basis of cDNA and genomic DNA (gDNA) from PC3 and DU145 cell lines by PCR. The results gave the information that circITGA7 could only be amplified by divergent primers in cDNA, instead of in gDNA (Figure 1(c)). Then, the RNase R assays illustrated that circITGA7 was more resistant to RNase R than linear mRNA (Figure 1(d)).

Subsequently, the expression levels of circITGA7 in 28 cases of PCa tissues and corresponding adjacent normal tissues were detected by qRT-PCR and the results demonstrated that circITGA7 was significantly downregulated in PCa tissues than that in normal tissues (Figure 1(e)). However, the expression level of circITGA7 was not remarkably correlated with pathological stage, Gleason score, and blood PSA level (Supplementary Table 1). Similarly, the expression of circITGA7 in PCa cells (PC3, LNCaP, and DU145) was greatly lower than that in normal prostate epithelial cells (RWPE-1) (Figure 1(f)). After that, in order to understand the subcellular localization of circITGA7, we conducted FISH experiment in PC3 cells and the results displayed that circITGA7 was mainly distributed in the cytoplasm (Figure 1(g)).

### 3.2. The Overexpression of circITGA7 Inhibits the Proliferation of PCa Cells *In Vivo* and *In Vitro*

**Figure 2:**
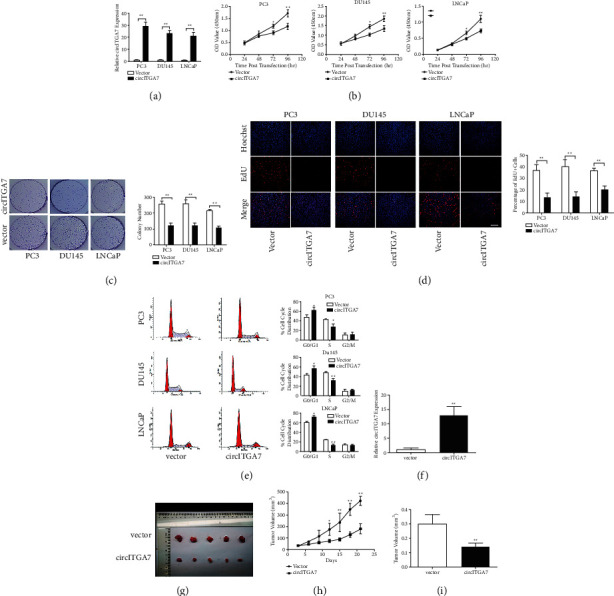
The enforced expression of circITGA7 inhibits cell proliferation *in vitro* and *in vivo*. (a) The expression levels of circITGA7 in PCa cells stably transfected with circITGA7 overexpression vector or negative control vector were detected by qRT-PCR. (b) CCK-8 assay was used to detect the growth rate of transfected PCa cells. (c, d) The cell proliferation was measured by the colony formation and EdU assays. Scale bar, 100 *μ*m. (e) The cell cycle was analyzed by flow cytometry analysis. (f) The expression of circITGA7 in xenograft tumors was detected. (g–i) Xenograft tumors derived from circITGA7 group showed a smaller volume and lower weight than those derived from empty vector group. Data were presented as mean ± SD of three independent experiments. Statistically significant differences were indicated:  ^*∗*^*P* < 0.05,  ^*∗*^ ^*∗*^*P* < 0.01.

In order to investigate the functions of circITGA7 in PCa, the circITGA7 overexpression vector was transfected into PCa cells. As shown in Figure 2(a), the expression level of circITGA7 was significantly increased after transfection. Afterwards, we performed CCK-8, colony formation, EdU, and flow cytometry analysis. CCK-8 assay revealed that the cell growth rate was inhibited after the overexpression of circITGA7 (Figure 2(b)). Similarly, colony formation assay and EdU assay exhibited that circITGA7 inhibited the proliferation of PCa cells (Figures 2(c) and 2(d)). Besides, flow cytometry analysis showed that overexpression of circITGA7 induced cell cycle arrest at G0/G1 phase (Figure 2(e)). To further explore the effects of circITGA7 on cell proliferation *in vivo*, PC3 cells stably transfected with circITGA7 were injected into nude mice and the expression of circITGA7 in xenograft tumors was detected (Figure 2(f)). As shown in Figures 2(g)–2(i), compared with the control vector group, the growth rate and tumor weight of the xenograft tumor in circITGA7 group were significantly decreased. Collectively, our results showed that circITGA7 could obviously inhibit PCa cell proliferation *in vivo* and *in vitro*.

### 3.3. Knockdown of circITGA7 Promotes PC Cell Proliferation

**Figure 3:**
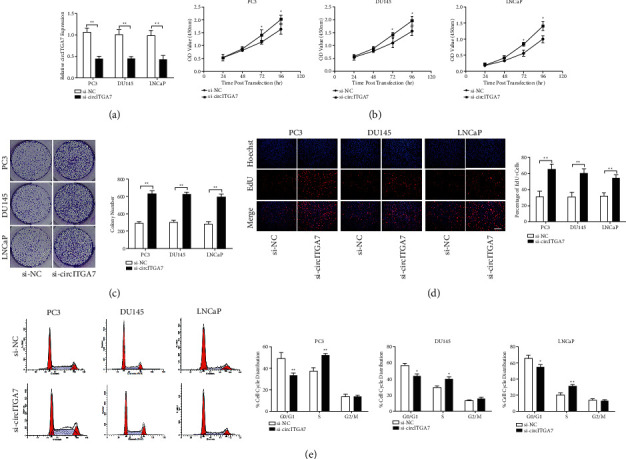
The knockdown of circITGA7 promotes the proliferation of PCa cells. (a) qRT-PCR assay was used to measure the efficiency of small interfering RNAs. (b) CCK-8 assay confirmed that the cell growth rate was increased after the knockdown of circITGA7. (c, d) Colony formation and EdU assays were used to detect cell proliferation after the knockdown of circITGA7. Scale bar, 100 *μ*m. (e) Flow cytometry analysis revealed circITGA7 knockdown promoted the cell transition from G1 phase to S phase. Data were presented as mean ± SD of three independent experiments. Statistically significant differences were indicated:  ^*∗*^*P* < 0.05,  ^*∗*^ ^*∗*^*P* < 0.01.

To further explore the effects of circITGA7 on PCa cell proliferation, we constructed small interfering RNA to knock down circITGA7 expression (Figure 3(a)). As shown in Figure 3(b), CCK-8 assay showed that the knockdown of circITGA7 significantly increased the cell growth rate of PCa cells compared with the negative control. Consistently, the following colony formation and EdU assays showed that the proliferation ability of PCa cells was increased after the knockdown of circITGA7 (Figures 3(c) and 3(d)). Moreover, flow cytometry analysis revealed that circITGA7 knockdown could accelerate the cell transition from G1 to S phase (Figure 3(e)).

### 3.4. circITGA7 Sponges miR-370-3p in PCa Cells

**Figure 4:**
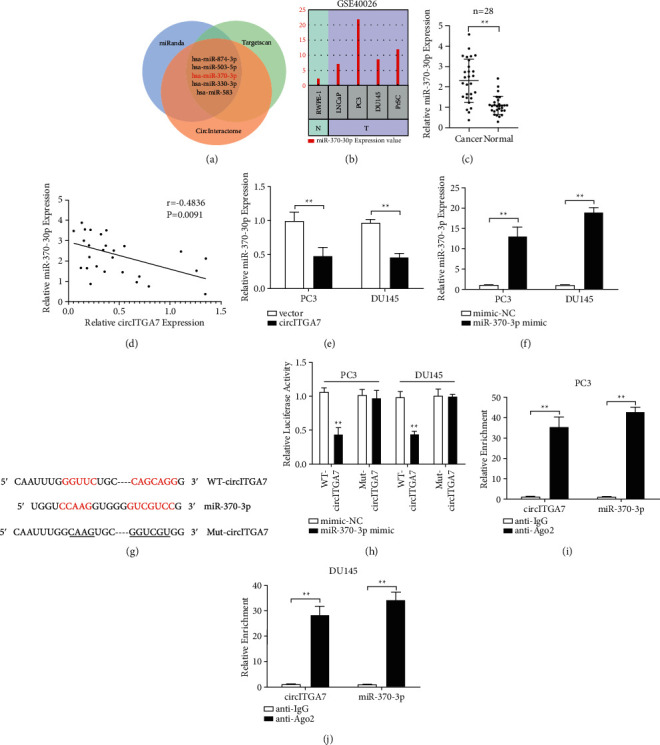
circITGA7 sponges miR-370-3p in PCa cells. (a) Bioinformatics analysis with miRanda, TargetScan, and CircInteractome. (b) The GEO dataset analysis showed the expression of miR-370-3p in PCa cells and normal prostate epithelial cells. (c) The expression level of miR-370-3p in 28 pairs of PCa and paracancerous tissues was detected. (d) Evaluating the correlation of circITGA7 and miR-370-3p expression in PCa tissues by Pearson correlation coefficient. (e) miR-370-3p was downregulated after the overexpression of circITGA7. (f) qRT-PCR was used to detect the relative expression of miR-370-3p in cells transfected with miR-370-3p mimic or negative control. (g) Schematic representation of the binding sites of miR-370-3p with circITGA7. (h) Relative luciferase activity was determined by dual‐luciferase reporter assay in PCa cells cotransfected with miR-370-3p mimic and WT‐circITGA7 or Mut‐circITGA7. (i, j) Interaction between miR-370-3p and circITGA7 was further confirmed by RIP assay. Data were presented as mean ± SD of three independent experiments. Statistically significant differences were indicated:  ^*∗*^ ^*∗*^*P* < 0.01.

Acting as highly efficient microRNA sponges to regulate protein-coding genes is an important way for circRNAs to exert its function. In order to elucidate the underlying mechanisms of circITGA7 regulating PCa progression, we performed bioinformatics analysis with miRanda, TargetScan, and CircInteractome and predicted five microRNAs that might target circITGA7 (Figure 4(a)). The analysis of GEO data GSE40026 suggested that the expression level of miR-370-3p in PCa cells was extremely higher than that in normal epithelial cells (Figure 4(b)). Moreover, miR-370-3p was reported to promote the proliferation of PCa [17]. Therefore, miR-370-3p was chosen for further study. First, the expression level of miR-370-3p in PCa tissues was examined by qRT-PCR. As shown in Figure 4(c), the expression of miR-370-3p in PCa tissues was obviously increased compared with that in adjacent normal tissues. Then, Pearson correlation analysis illustrated that the expression level of miR-370-3p was negatively correlated with circITGA7 (Figure 4(d)). Additionally, the overexpression of circITGA7 remarkably reduced the level of miR-370-3p (Figure 4(e)).

To elucidate whether circITGA7 could directly bind miR-370-3p, we then performed dual-luciferase assay and the results showed that miR-370-3p mimic significantly reduced the luciferase activity of circITGA7 compared with miR‐NC, while miR-370-3p mimic had no significant effect on luciferase activity of circITGA7 with the mutated target site (Figures 4(f)–4(h)). The next RIP assay exhibited that circITGA7 and miR-370-3p were enriched in RNA complexes in the Ago2 group compared with the IgG group (Figures 4(i) and 4(j)).

### 3.5. P21^CIP1^ Is a Target of miR-370-3p in PCa Cells

**Figure 5:**
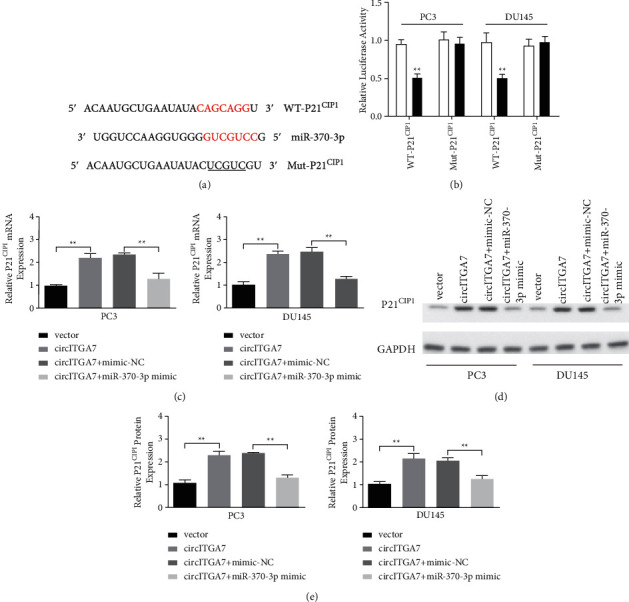
circITGA7 upregulates the expression level of P21^CIP1^ via miR‐370‐3p. (a) The binding sites of miR‐370‐3p in 3ʹ UTR of P21^CIP1^ were predicted by bioinformatics analysis. (b) Luciferase reporter assays were performed following the cotransfection of the cells with WT-P21^CIP1^ or Mut-P21^CIP1^ and miR‐370‐3p mimic or NC mimic. The mRNA (c) and protein levels (d, e) of P21^CIP1^ were examined after the cotransfection of circITGA7 overexpression plasmid and miR-370-3p mimic. Data were presented as mean ± SD of three independent experiments. Statistically significant differences were indicated:  ^*∗*^ ^*∗*^*P* < 0.01.

In order to further study the regulation mechanism of miR-370-3p, we predicted the downstream target of P21^CIP1^ with TargetScan databases. As shown in Figure 5(a), miR-370-3p may target P21^CIP1^, so luciferase plasmid with WT-P21^CIP1^ 3ʹUTR or Mut-P21^CIP1^ 3ʹUTR was constructed to further verify the relationship between miR-370-3p and P21^CIP1^. We observed that miR-370-3p mimic significantly inhibited the luciferase activity in cells that carry a luciferase plasmid containing WT-P21^CIP1^ 3ʹUTR, but not the luciferase plasmid containing Mut-P21^CIP1^ 3ʹUTR (Figure 5(b)). In addition, the mRNA and protein level of P21^CIP1^ increased significantly after the overexpression of circITGA7, while this effect was reversed by miR-370-3p mimic (Figures 5(c)–5(e)). Collectively, the above results indicated that circITGA7 regulated the expression of P21^CIP1^ via sponging miR-370-3p.

### 3.6. The Overexpression of miR-370-3p Eliminates the Inhibitory Effect of circITGA7 on PCa Cell Proliferation

**Figure 6:**
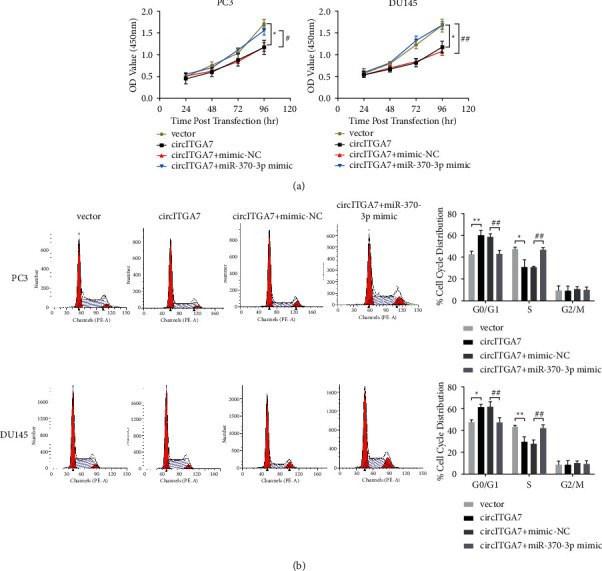
Transfection of miR-370-3p mimic reverses the inhibitory effect of circITGA7 on cell proliferation. (a) The cell growth rate was detected by CCK-8 assay after the cotransfection with circITGA7 overexpression plasmid and miR-370-3p mimic. (b) Cell cycle distributions were presented by flow cytometry analysis. Data were presented as mean ± SD of three independent experiments. Statistically significant differences were indicated:  ^*∗*^*P* < 0.05,  ^*∗*^ ^*∗*^*P* < 0.01, circITGA7 versus vector; ^#^*P* < 0.05, ^##^*P* < 0.01, circITGA7 + miR-370-3p mimic versus circITGA7 + mimic-NC.

To test whether circITGA7 inhibits the proliferation of PCa cells through miR-370-3p, we conducted the rescue assay. As shown in Figure 6(a), the enforced expression of circITGA7 significantly inhibited PCa cell proliferation, but this inhibition effect was eliminated after transfecting with miR-370-3p mimic. A similar phenomenon was found in subsequent flow cytometry analysis. Cell cycle was arrested in G0/G1 phase after the overexpression of circITGA7, but cells were accelerated into S phase by transfection of miR-370-3p mimic (Figure 6(b)). Together, these results indicated that circITGA7 contributed to cell proliferation inhibition by miR-370-3p/P21^CIP1^ axis.

## 4. Discussion

Recently, the role of circRNAs in cancer progression has attracted increasing attention and several circRNAs have been identified as potential biomarkers for cancer [18, 19]. It has been reported that CircSLC19A1 silencing inhibited PCa cell proliferation, migration, and invasion by regulating miR-326/MAPK1 axis [20]. Zhang et al. proposed that Circ_0057553 affected PCa cell viability, migration, invasion, apoptosis, and glycolysis through miR-515-5p/YES1 axis [21]. Shan et al. held the view that circFMN2 promoted the proliferation of PCa cells by facilitating DNA synthesis and inhibiting cell apoptosis [22]. These studies indicated that circRNAs played an important role in the development and progression of PCa.

In this study, we demonstrated that the expression of circITGA7 in PCa tissues and cells was significantly lower than that in normal prostate tissues and cells. However, the expression level of circITGA7 was not significantly correlated with pathological stage, Gleason score, and PSA level. Considering the small sample size of our study, this conclusion still needs to be further clarified with a larger sample size. Fang et al. reported that circITGA7 promoted the proliferation and metastasis of osteosarcoma cells [13], while Li et al. discovered that circITGA7 inhibited the growth and metastasis of colorectal cancer [11]. The two studies with different conclusions revealed the diversity of biological roles of circITGA7 in different tissues. In our present study, the loss- and gain-of-function assays confirmed that circITGA7 inhibited cell proliferation in PCa, but the effects of circITGA7 on cell invasion and migration are still unclear, which will be explored in our future studies.

Those circRNAs derived from protein encoding exons and primarily localized in the cytoplasm can function by sponging miRNAs [23]. Jin et al. revealed that circLMTK2 acted as a tumor suppressor in PCa via regulating the expression of microRNA-183 [24]. circAMOTL1 was found to serve as a competing endogenous RNA to prompt the expression of AMOTL1 through sponging miR-485-5p in cervical cancer [25]. Considering that circITGA7 was derived from exon 4 of ITGA7 gene and mainly located in the cytoplasm, we speculated that circITGA7 might act as a miRNA sponge, and then conducted miRNA-target analysis. Through overlapping screening of miRanda, TargetScan, and CircInteractome, combined with GEO data analysis and literature search, miR-370-3p was selected as the target of circITGA7 for the next step of research. miR-370-3p has been reported to be involved in a wide range of human diseases, such as acute pneumonia [26], endometriosis [27], hypoxia injury of myocardial cells [28], and especially cancers [29–31]. Through further mechanism research, we revealed that circITGA7 could bind with miR-370-3p and negatively regulate miR-370-3p expression in PCa cells.

P21^CIP1^, encoded by CDKN1A, is one of the key factors in cell cycle regulation. Several studies have pointed out that P21^CIP1^ is involved in the G1-S phase cell cycle transition [32–34]. Wu et al. have confirmed that miR-370-3p can promote G1-S phase transition of prostate cancer cells partly by downregulating P21^CIP1^ [17]. Besides, P21^CIP1^ was predicted as a target of miR-370-3p by TargetScan. Therefore, P21^CIP1^ was selected as the downstream target of miR-370-3p for further study. In our study, the overexpression of circITGA7 could upregulate the expression of P21^CIP1^ and induce G0/G1 cell cycle arrest, while this effect was reversed by transfecting of miR-370-3p mimic. Our results preliminarily expounded the mechanism of circITGA7 that regulated the proliferation of PCa cells.

## 5. Conclusions

In conclusion, our study demonstrated that circITGA7 was downregulated in PCa tissues and cell lines for the first time. circITGA7 inhibited the proliferation of PCa cells and induced G0/G1 phase arrest. Mechanistically, functioning as a sponge for miR-370-3p, circITGA7 increased P21^CIP1^ expression. Our data enhanced our understanding of circRNA biology and proposed a novel circRNA/miRNA/mRNA regulatory network for the management of PCa.

## Data Availability

The datasets and supporting data are available from the corresponding author upon reasonable request.

## Supplementary Materials

Supplementary Materials Supplementary Table 1: clinicopathologic characteristics of patient samples and the expression of circITGA7 in PCa. The specimens were classified according to the 2017 version of American Joint Committee on Cancer. Supplementary Table 2: the primer and siRNA sequences used in the study.
