# Supplementary material for: circITGA7 Acts as a miR-370-3p Sponge to Suppress the Proliferation of Prostate Cancer
Source: J Oncol. 2021 Dec 31;2021:8060389. doi: 10.1155/2021/8060389 (PMC8741341; doi:10.1155/2021/8060389)
Supplement: Supplementary Materials — Supplementary Table 1: clinicopathologic characteristics of patient samples and the expression of circITGA7 in PCa. The specimens were classified according to the 2017 version of American Joint Committee on Cancer. Supplementary Table 2: the primer and siRNA sequences used in the study. [file 8060389.f1.docx]

**Table 1. Clinicopathologic characteristics of patient samples and the expression of circITGA7 in PCa.**

|  |  |  | Expression of circITGA7 | |  |
| --- | --- | --- | --- | --- | --- |
| Parameters | Group | Cases | Low | High | p-value |
| Age (year) | >66 | 13 | 9 | 4 | >0.9999 |
|  | ≤66 | 15 | 10 | 5 |  |
| Pathological stage | ≤T2b | 16 | 9 | 7 | 0.2232 |
|  | >T2b | 12 | 10 | 2 |  |
| Gleason score | ≤7 | 12 | 6 | 6 | 0.1139 |
|  | >7 | 16 | 13 | 3 |  |
| PSA (ng/ml) | ≤20 | 17 | 11 | 6 | >0.9999 |
|  | >20 | 11 | 8 | 3 |  |
| Total |  | 28 | 19 | 9 |  |

The specimens were classified according to the 2017 version of American Joint Committee on Cancer. P < 0.05 represents statistical significance (Chi-square test).

**Table 2. Sequences in the study.**

| Oligonucleotides | Sequence (5ʹ -3ʹ) |
| --- | --- |
| circITGA7 Forward | CCCCAAGGCCATGAACAATT |
| circITGA7 Reverse | TCCCCACCATCCAACTCATC |
| ITGA7 Forward | CTGACTCCATGTTCGGGATCA |
| ITGA7 Reverse | CACCTGTGAAGGTTTGGCG |
| P21^cip1^ Forward | TGTCCGTCAGAACCCATGC |
| P21^cip1^ Reverse | AAAGTCGAAGTTCCATCGCTC |
| miR-370-3p Forward | TGTAACCAGAGAGCGGGATGT |
| miR-370-3p Reverse | TTTTGGCATA ACTAAGGCCGAA |
| GAPDH Forward | CTTCTTTTGCGTCGCCAGCC |
| GAPDH Reverse | TTCTCAGCCTTGACGGTGCC |
| U6 Forward | CTCGCTTCGGCAGCACA |
| U6 Reverse | AACGCTTCACGAATTTGCGT |
| si-circITGA7 sense | CCUAUAAUUGGAAGGACCUTT |
| si-circITGA7 antisense | AGGUCCUUCCAAUUAUAGGTT |
